# A Homozygous Dab1^−/−^ Is a Potential Novel Cause of Autosomal Recessive Congenital Anomalies of the Mice Kidney and Urinary Tract

**DOI:** 10.3390/biom11040609

**Published:** 2021-04-20

**Authors:** Anita Racetin, Natalija Filipović, Mirela Lozić, Masaki Ogata, Larissa Gudelj Ensor, Nela Kelam, Petra Kovačević, Koichiro Watanabe, Yu Katsuyama, Mirna Saraga-Babić, Merica Glavina Durdov, Katarina Vukojević

**Affiliations:** 1 Department of Anatomy, Histology and Embryology, University of Split School of Medicine, 21000 Split, Croatia; amuic@mefst.hr (A.R.); natalija.filipovic@mefst.hr (N.F.); mirelalozic3@gmail.com (M.L.); larissagudeljensor@yahoo.co.uk (L.G.E.); nelakelam6@gmail.com (N.K.); msb@mefst.hr (M.S.-B.); 2 Department of Medical Genetics, School of Medicine, University of Mostar, 88000 Mostar, Bosnia and Herzegovina; petra.kovacevic110@gmail.com; 3 Division of Anatomy, Faculty of Medicine, Tohoku Medical and Pharmaceutical University, Sendai, Miyagi 981-8558, Japan; mogata@tohoku-mpu.ac.jp; 4 Department of Anatomy, Shiga University of Medical Science, Ötsu 520-2192, Japan; ds111799@g.shiga-med.ac.jp (K.W.); kats@belle.shiga-med.ac.jp (Y.K.); 5 Department of Pathology, University Hospital of Split, 21000 Split, Croatia; merigdst@yahoo.co.uk

**Keywords:** *yotari*, kidney function, postnatal kidney development, immunofluorescence staining, transmission electron microscopy

## Abstract

This study aimed to explore morphology changes in the kidneys of *Dab1^−/−^* (*yotari)* mice, as well as expression patterns of reelin, NOTCH2, LC3B, and cleaved caspase3 (CASP3) proteins, as potential determinants of normal kidney formation and function. We assumed that *Dab1* functional inactivation may cause disorder in a wide spectrum of congenital anomalies of the kidney and urinary tract (CAKUT). Animals were sacrificed at postnatal days P4, P11, and P14. Paraffin-embedded kidney tissues were sectioned and analyzed by immunohistochemistry using specific antibodies. Kidney specimens were examined by bright-field, fluorescence, and electron microscopy. Data were analyzed by two-way ANOVA and t-tests. We noticed that *yotari* kidneys were smaller in size with a reduced diameter of nephron segments and thinner cortex. TEM microphotographs revealed foot process effacement in the glomeruli (G) of *yotari* mice, whereas aberrations in the structure of proximal convoluted tubules (PCT) and distal convoluted tubules (DCT) were not observed. A significant increase in reelin expression, NOTCH2, LC3B and cleaved CASP3 proteins was observed in the glomeruli of *yotari* mice. Renal hypoplasia in conjunction with foot process effacement and elevation in the expression of examined proteins in the glomeruli revealed CAKUT phenotype and loss of functional kidney tissue of *yotari.*

## 1. Introduction

*Yotari* mutant mice, obtained by spontaneous acquisition of a mutation in the *Dab1* gene, exhibit histological abnormalities in the central nervous system [1,2], very similar to those of *reeler* (*reelin^−/−^*) mice, suggesting that reelin and DAB1 belong to the same signaling pathway [1,2,3,4]. The aberrations in the reelin/DAB1 pathway have been reported to be associated with various psychiatric disorders [5,6,7].

Interestingly, besides the central nervous system, the presence of DAB1 and reelin proteins has also been confirmed in the peripheral nervous system and some extraneural tissues. The presence of DAB1 has been found in mouse podocytes [8] and human fetal kidneys [9], while reelin expression has been reported during mouse and human fetal development, as well as in some endothelial cells along blood vessels in the adult mouse [9,10,11].

The canonical reelin/DAB1 pathway may trigger distinct downstream proteins, including NOTCH2 receptors, which play critical roles in cell-fate decisions and differentiation during kidney development [12,13,14]. NOTCH2 receptors are required to form proximal tubules and podocytes [13], but once nephron maturation is achieved, this pathway is mostly silenced [15]. In conditions of acute kidney diseases, increased expression of NOTCH2 may potentially contribute to regeneration, while sustained expression is causally associated with interstitial fibrosis and glomerulosclerosis [15]. Downregulation of NOTCH signaling can greatly diminish autophagy and cause podocyte differentiation impairment during development [16].

Consequently, dysfunctional podocytes play a crucial role in glomerular disease, especially in human idiopathic nephrotic syndrome [17,18] and in minimal change nephrotic syndrome (MCNS) [19]. Hence, autophagy maintains cellular homeostasis under normal physiological conditions, whereas in pathological conditions, it can progress into autophagic death [20]. A widely used autophagy biomarker is LC3B, a structural protein of autophagosomal membranes [21]. Complex crosstalk between autophagy and apoptosis also contributes to the maintenance of homeostatic balance as a response to the cell’s microenvironment. It is well-known that apoptotic events increase in number during kidney development [22] and greatly diminish once maturation is over, except in conditions of renal injury [23]. Undeniably, apoptosis is a complex mechanism regulated through the interplay of several pathways but mostly finishes with activation of caspase3 (CASP3), whose activation is one of the most often used markers for apoptosis detection [23].

In this report, we aimed to analyze how *Dab1* gene functional silencing influences kidney morphology and the expression and localization of reelin, NOTCH2, LC3B, and cleaved CASP3 proteins in postnatal mice kidneys. We assume that these proteins are expressed in the postnatal mouse kidney, and their functional interplay contributes to the maintenance of their structure and function. In addition, as the presence of DAB1 has been confirmed during fetal human kidney development [9], it could be presumed that its inactivation may cause disorder in a wide spectrum of congenital anomalies of the kidney and urinary tract (CAKUT).

## 2. Materials and Methods

### 2.1. Sample Collection

As *Dab1* null conventional mutants, we used *yotari (Dab1^−/−^)* mice previously describes by Howell et al. [24]. PCR primers used for genotyping of the mice were *yotari*: GCCCTTCAGCATCACCATGCT and CAGTGAGTACATATTGTGTGAGTTCC, wild-type of Dab1 locus: GCCCTTCAGCATCACCATGCT and CCTTGTTTCTTTGCTTTAA-GGCTGT [5]. C57BL/6 N mice were raised and group-housed in standard polycarbonate cages (including at least one of each genotype) with ad libitum access to food and water in a temperature-controlled (23 ± 2 °C) room with a 12 h light/dark cycle.

On postnatal days 4, 11 and 14 (P4, P11, and P14), mice were deeply anesthetized with pentobarbital and transcardially perfused for 10 min with phosphate buffer saline (PBS, pH 7.2) and 4% paraformaldehyde (PFA) in 0.1 M PBS. Kidneys were removed and fixed with 4% PFA in 0.1 M PBS overnight for conventional histological analyses (hematoxylin–eosin (H&E), immunohistochemical and immunofluorescence staining) and in a 2% PFA + 2.5% glutaraldehyde (GA) mixture for electron microscope examination. After fixation, tissue was prepared for further histological examination, as we have described previously [25,26].

### 2.2. Immunofluorescence and Immunoperoxidase Staining

After deparaffinization and rehydration, sections were heated for 20 min in the 0.01 M citrate buffer (pH 6.0) in a water steamer and, afterward, cooled down to room temperature. Blocking buffer (ab 64226, Abcam, Cambridge, UK) was applied for 30 min to exclude unspecific staining. Sections were then incubated in a humidity chamber overnight with primary antibodies (Table 1). After washing in PBS, secondary antibodies (Table 1) were applied for one hour and washed in PBS again. Then, nuclei were stained with 4′6′-diamidino-2-phenylindole (DAPI) for 2 min, washed in PBS, and coverslipped. We performed the preadsorption test so that each primary antibody was used with the corresponding peptide and apply their combination to the sections. The results showed no antibody staining. In addition, controls with the omission of the primary antibody to determine levels of nonspecific binding of secondary antibodies were performed.

For immunoperoxidase staining, rabbit polyclonal anti-CASP3 (1:100 dilutions, ab13847, Abcam, Cambridge, UK) was applied as a primary antibody for one hour in a humidity chamber after the treatment of the section with 0.1% H_2_O_2_. After washing in PBS, secondary detection was performed using the link and streptavidin peroxidase, each for fifteen minutes (DakoCytomation, CA 93013 USA, LOT 03477) and diaminobenzidine (Dako, CA 93013 USA, LOT 10051369), carefully controlled to avoid background overstaining. Nuclei were stained with hematoxylin, rinsed in tap water for 10 min, briefly dehydrated in ascending series of ethanol solutions, and coverslipped.

### 2.3. Tissue Preparation for Transmission Electron Microscope (TEM)

After fixation with a mixture of 2% PFA and 2.5% GA in 0.1 M PBS for 2 h, the samples were washed with PBS and postfixed in an aqueous solution of 2% osmium tetroxide for 2 h. All samples were washed twice in PBS, dehydrated in ascending grades of ethanol and embedded in Epon 812 (TAAB Laboratories Equipment, Reading, UK). Serial sections (70 nm in thickness) were cut on an Ultracut UCT ultramicrotome (Leica Microsystems, Wetzlar, Germany) and stained with 1% uranyl acetate and lead citrate.

### 2.4. Data Acquisition and Analysis

The analysis was performed with an epifluorescence microscope (Olympus BX51, Tokyo, Japan) equipped with a DP71 digital camera (Olympus), a JEM 1400 transmission electron microscope (JEOL, Tokyo, Japan) operated at 80 kV and photographed with a JEOL charge-coupled device (CCD) camera system (Advanced Microscopy Techniques, Danvers, MA, USA) and lastly, a bright-field microscope (BX40, Olympus, Tokyo, Japan). All analyzed images were processed with ImageJ software and Adobe Photoshop (Adobe, San Jose, CA, USA). Per examined group, we used three to four animals. All collected kidneys were cut at 5 µm thick sections, and maximum kidney length determined by using these samples was reported. Mean proximal convoluted tubules (PCT), distal convoluted tubules (DCT) and glomeruli diameters were determined by averaging 100 structure diameters per analyzed sample. As nephron segments have irregular shapes, we took the largest diameter of every examined segment as representative. The staining intensity was semiquantitatively evaluated at four degrees: the absence of any reactivity (−), mild reactivity (+), moderate reactivity (++), and strong reactivity (+++) (Table 2). The number of DAB1, reelin, NOTCH2, LC3B, and cleaved CASP3 immunoreactive cells was counted and expressed as a percentage of total cells. For each sample, we analyzed twenty PCT, DCT, and G at ×40 objective magnification. We averaged the number of positive cells per group. Any level of nuclear, cytoplasmic, or membrane staining was regarded as positive. Three investigators analyzed the images independently. Interrater agreement was tested with interclass correlation analysis, which yielded a coefficient >0.75, indicating excellent agreement [27].

### 2.5. Statistical Analysis

A two-tailed t-test was performed to analyze the differences in the average diameter between PCT, DCT and G of wild-type and *yotari* animals. The diameter was presented as mean ± standard deviation (SD). The level of significance was set at *p* < 0.05. A two-way ANOVA test followed by Tukey’s multiple comparison test was used to examine the differences in the percentage of positive cells between PCT, DCT, and glomeruli at all-time points. The percentage of positive cells was expressed as the mean ± standard error of the mean (SEM). The level of significance was set at *p* < 0.05. The analysis was performed in GraphPad software (GraphPad Software, La Jolla, CA, USA).

## 3. Results

### 3.1. Hematoxylin-Eosin Staining (H&E) and Measurement of Kidney Diameter

**Figure 1:**
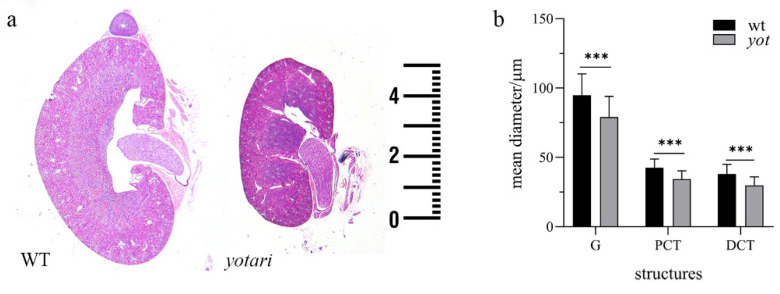
Light microscopy examination of the wild-type and *yotari* kidneys at 4P. (**a**) Comparison of wild-type (left) and *yotari* (right) kidney size. (**b**) Mean diameters of glomeruli (G), convoluted tubules (PCT) and distal convoluted tubules (DCT) in the kidneys of *yotari* animals were significantly smaller compared to wild-type animals. Data presented as the mean ± SD (vertical line). Significant differences were indicated by *** *p* < 0.0001 (two-tailed *t*-test). Per each sample, 100 PCTs, DCTs, and glomeruli were assessed. Scale bar is 1 mm, refers to images in (**a**).

H&E staining of midsagittal sections of the kidneys at all examined timepoints highlights the small kidney phenotype of *yotari* mice compared to wild-type (Figure 1a). At 4P mean kidney diameter of *yotari* animals was around 55 µm than around 75 µm. In addition, this difference was noticeable at later time points due to the slower growth of the kidneys in *yotari* compared to the progressive growth of the kidneys in wild-type animals. To determine whether the reduction in overall kidney size was caused by a decrease in nephron segment size, we averaged the diameter of PCT, DCT and G per group. Indeed, there was a decrease in mean diameter G, PCT and DCT in the *yotari* group compared to wild-type (Figure 1b, *p* < 0.05). Additionally, H&E staining revealed thinner cortex and renal pelvis extension in *yotari* animals and slightly diffusely dilated DCT at 14P. The basic structure and pattern of glomerular maturation are the same in both examined animal groups.

### 3.2. Descriptive Histological Analysis Based on TEM Microphotographs

**Figure 2:**
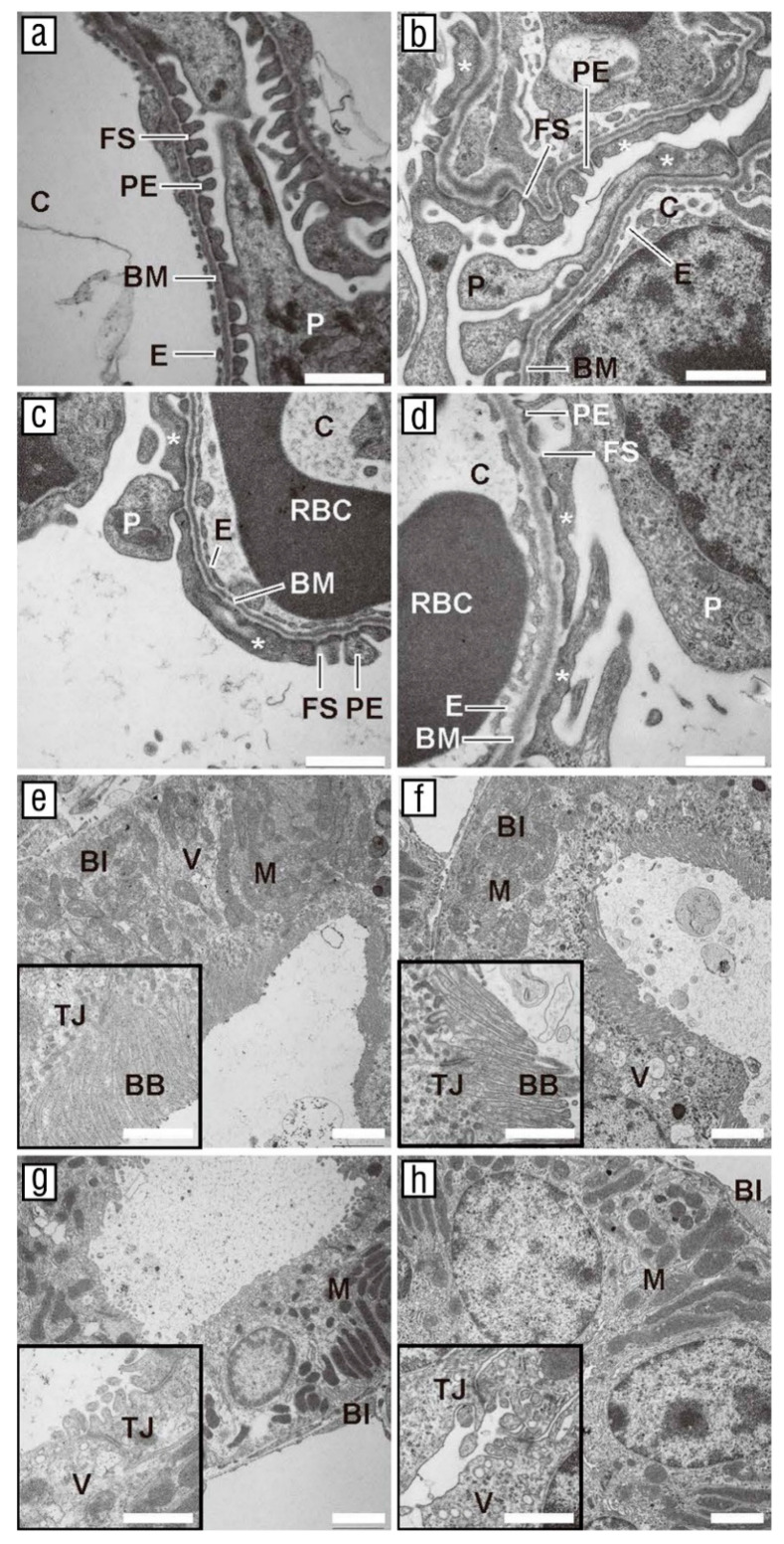
Transmission electron microscope (TEM) microphotographs of wild-type and *yotari* kidneys. Glomeruli of wild-type animals (**a**) showed a normally developed filtration barrier with fenestrated endothelium (E) of the capillary (C), basement membrane (BM), podocytes (P) with pedicles (PE), and filtration slits (FS). In the kidneys of *yotari* animals, effacement of the pedicles and lack of filtration slits could be noticed (asterisks, **b**–**d**). No abnormalities were noticed in the proximal convoluted tubules (PCT) or distal convoluted tubules (DCT) of *yotari* mice compared to the wild-type animals (**e**–**h**). In the PCT, the cytoplasm was abundant, containing well-distinguished nuclei (N), elongated mitochondria (M), basal infoldings (BI), and many tubular pits (TP) between microvilli, which formed a brush border (BB) at the apical membrane (**e**,**f**). Higher magnification revealed the apical surface of PCT epithelial cells containing long microvilli to form the brush border and tight junctions (TJ) between the luminal cell borders of neighboring tubular epithelial cells (**e**,**f**). On the other hand, the apical surface of DCT epithelial cells containing few short microvilli and numerous vesicles (**g**,**h**). The scale bar in images (**a**–**d**) and insets (**e**–**h**) is 1 µm; the scale bar in images (**e**–**h**) is 2 µm.

On microphotographs obtained by TEM, glomeruli of wild-type mice showed the typical appearance of all parts of the filtration barrier, which were normally developed and recognizable (Figure 2a). Podocytes, podocyte foot processes and pedicles, filtration slits, glomerular basement membranes, and the capillary lumen with the fenestrated endothelium were easily distinguishable (Figure 2a). On the other hand, in the glomeruli of *yotari* mice, progressive podocyte damage with foot process effacement and lack of filtration slits could be observed (Figure 2b–d). These ultrastructural changes were observed in all kidneys examined and involved most of the examined glomeruli. No abnormalities were noticed in the PCT or DCT of *yotari* mice than wild-type animals (Figure 2e–h). In the PCT, cell interfaces were defined poorly because of the irregularity of cell membranes and cell interdigitation with their neighbors. The cytoplasm was abundant, containing well-distinguished nuclei, elongated mitochondria, basal infoldings, and many tubular pits between microvilli, which formed a brush border at the apical membrane. Higher magnification revealed the apical surface of PCT epithelial cells containing long microvilli to form the brush border and tight junctions between the luminal cell borders of neighboring tubular epithelial cells (Figure 2e,f). On the other hand, the apical surface of DCT epithelial cells contained few short microvilli and numerous vesicles (Figure 2g,h).

### 3.3. Localization and Colocalization of Reelin and DAB1

**Figure 3:**
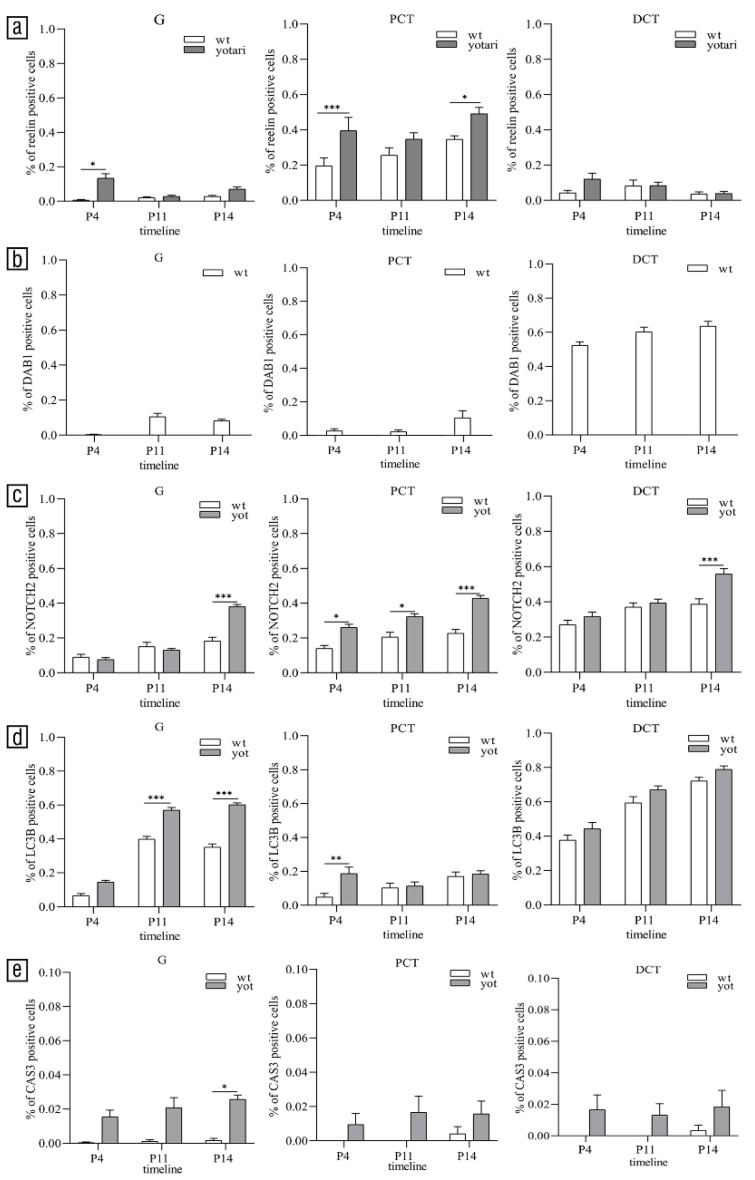
The distribution of the percentages of reelin (**a**), DAB1 (**b**), NOTCH2 (**c**), LC3B (**d**) and cleaved CASP3-positive (**e**) cells in the glomeruli (G), proximal convoluted tubules (PCT), distal convoluted tubules (DCT) of wild-type and *yotari* kidneys. Data presented as the mean ± SEM (vertical line). Significant differences were indicated by * *p* < 0.05, ** *p* < 0.001, *** *p* < 0.0001 (Two-way ANOVA followed by Tukey’s multiple comparison test). At each time point, 20 PCTs, DCTs, and glomeruli were assessed.

**Figure 4:**
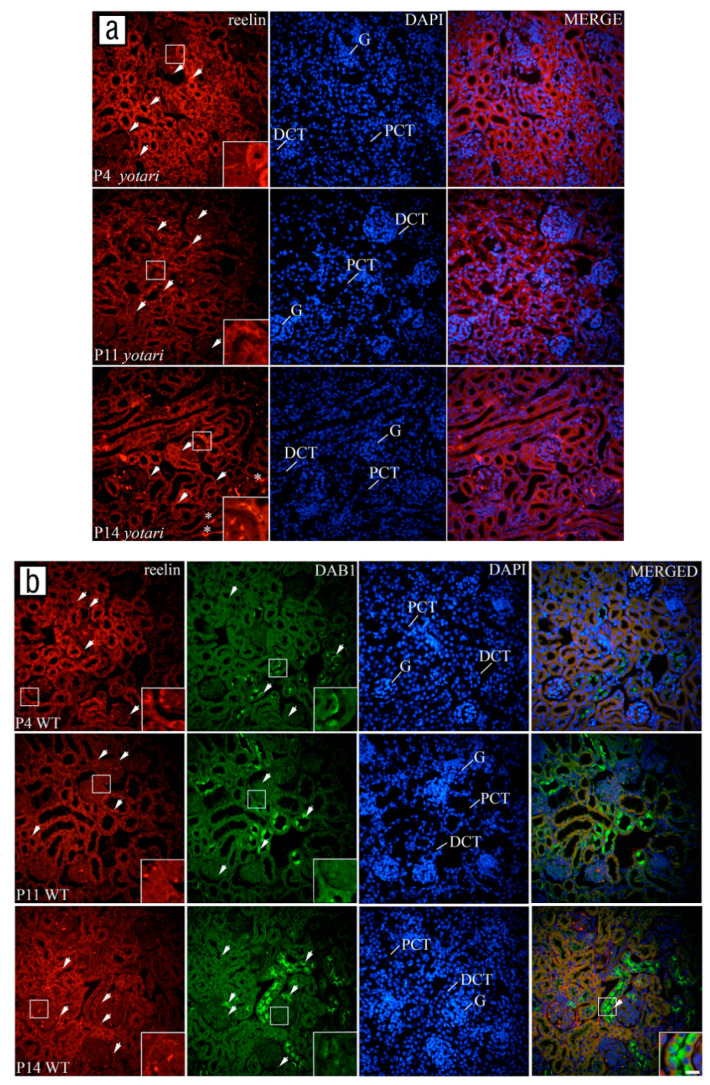
Immunofluorescence staining of postnatal *yotari* kidneys with reelin marker (**a**) and double immunofluorescence staining of postnatal wild-type kidneys with DAB1 and reelin markers (**b**). (**a**,**b**) Nuclear DNA DAPI staining merged with DAB1, and reelin immunofluorescence is shown in parallel (merge). Observed time points were P4, P11, P14. Expression of the examined markers in the glomeruli (G), proximal convoluted tubules (PCT) and distal convoluted tubules (DCT) is marked with the arrows, while asterisks are indicating expression of the reelin in the extracellular matrix (**a**). Inserts show the most prominent expression in the image. DAPI nuclear staining revealed poor colocalization of DAB1 and reelin, mostly in the DCT (arrowhead). Scale bar is 20 µm and refers to all images.

Reelin was weakly expressed with mild to moderate reactivity (Table 2) in the glomeruli and DCT of all examined animals at all observed time points (*p* < 0.05, Figure 3a). Only in the PCT of *yotari* mice was the percentage of positive cells significantly higher than wild-type animals (*p* < 0.05, Figure 3a). In the glomerular cells, the localization of staining was perinuclear, while in the PCT and DCT, it was scattered throughout the cytoplasm (Figure 4a,b).

There was almost no immune reactivity of DAB1 in glomeruli and PCT of wild-type animals at P4, while the percentage of positive cells with mild reactivity (Table 2) significantly increased at P11 and P14 (*p* < 0.05, Figure 3b). In the DCT, DAB1 was expressed mostly at the apical and lateral parts of cell membranes (Figure 4b) with strong reactivity (Table 2). The percentage of positive cells was significantly higher than in previous structures, around 60% (Figure 3b), especially in the macula densa (Figure 4b).

There was almost no colocalization of the DAB1 and reelin except in the DCT of wild-type animals at P14 (arrowhead, Figure 4b).

### 3.4. Spatial and Temporal Expression Patterns of NOTCH2 and LC3B

**Figure 5:**
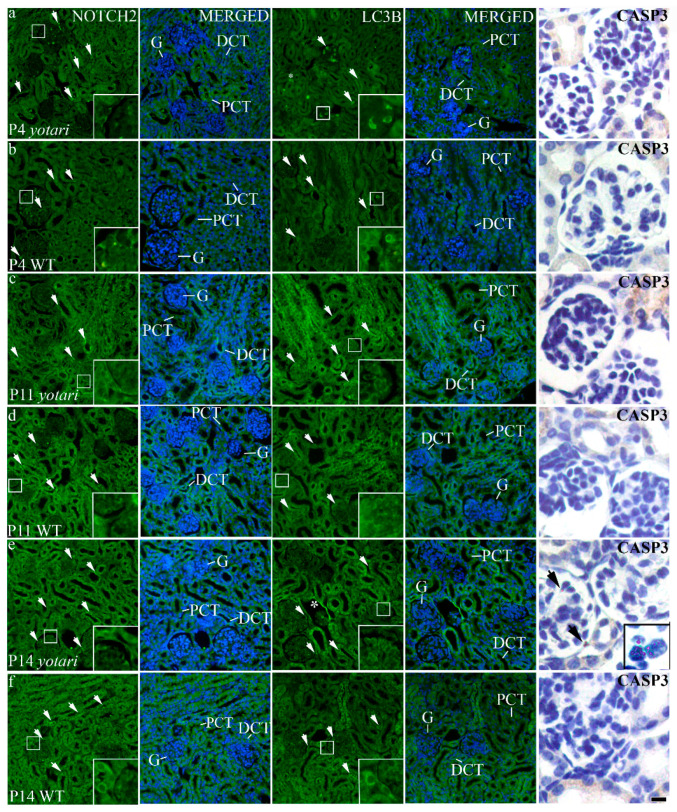
Immunofluorescence staining of postnatal wild-type and *yotari* kidneys with NOTCH2 and LC3B markers and immunoperoxidase staining with cleaved caspase3 (CASP3) marker. (**a**–**f**) Nuclear DNA DAPI staining is merged with NOTCH2 and LC3B. Observed time points were P4, P11, P14. Expression of the NOTCH2 and LC3B markers in the glomeruli (G), proximal convoluted tubules (PCT) and distal convoluted tubules (DCT) is marked with the arrows. Especially strong LC3B reactivity can be observed in the Bowman capsule of decaying glomeruli (asterisk, e). Inserts show the most prominent expression in the image. CASP3 was poorly expressed in all observed time points. The only significant expression was in the glomeruli of *yotari* mice on P14 (arrows). Scale bar is 20 µm and refers to all images.

The percentage of NOTCH2-positive cells was less than 20% in the glomeruli of both animal genotypes at all observed time points. Only at P14 percentage of positive cells was significantly higher in the glomeruli of *yotari* animals than wild-type animals (*p* < 0.05, Figure 3c). The staining intensity was mild to moderate (Table 2), mostly located perinuclearly (Figure 5a–f). In the PCT and DCT of both animal genotypes percentage of positive cells increased through time. At P14, the percentage of positive cells was significantly higher in PCT and DCT of *yotari* animals than PCT and DCT of wild-type animals (*p* < 0.05, Figure 3c). Signal intensity at all observed time points was mild to moderate (Table 2) and scattered throughout the cytoplasm (Figure 5a–f).

In the glomeruli and DCT of both animal genotypes, the percentage of LC3B-positive cells increased through time (*p* < 0.05, Figure 3d). At P11 and P14, the percentage of positive cells was significantly higher in the glomeruli of *yotari* animals than the glomeruli of wild-type animals (*p* < 0.05, Figure 3d). In the glomeruli of *yotari* animals staining intensity was mostly moderate to strong (Table 2) located in the perinuclear and nuclear space (Figure 5a–f), at all observed time points, while in the glomeruli of wild-type mice, the intensity was mostly moderate (Table 2). PCT of *yotari* and wild-type animals contained around 20% of positive cells at all observed time points (*p* < 0.05, Figure 3d).

### 3.5. Cleaved CASP-3 Expression

There was almost no expression of the activated CASP-3 in the kidneys of wild-type animals at all observed time points. In the PCT and DCT of *yotari* mice, several individual cells were CASP-3-positive (Figure 3e). Only in the glomeruli of *yotari* kidneys at P14 (Figure 5e), the percentage of positive cells significantly increased compared to kidneys of wild-type mice (*p* < 0.05, Figure 3e).

## 4. Discussion

Kidney morphogenesis and development are complex processes precisely coordinated through the interplay of a large number of genes. Congenital anomalies of the kidney and urinary tract (CAKUT) are the most common birth defect that constitutes 23% of all such defects and represents the leading cause of end-stage renal disease in children [28,29]. Single gene disorders may be the primary source of CAKUT, and until now, a mutation in more than 20 genes has been identified as the cause of CAKUT [30]. As our previous work showed great expression of DAB1 during normal fetal human kidney development [9], we assumed that DAB1 might play a significant role during mammalian kidney development. To prove our hypothesis, we investigated the kidney morphology and expression patterns of reelin, NOTCH2, LC3B, and activated CASP3 proteins in *Dab1* knockout mice.

Sectioning through the *yotari* mice kidneys revealed a thinner cortex and a significantly smaller mean kidney and PCT, DCT and G diameters compared to wild-type animals. The decrease in kidney size caused by insufficient nephron endowment is known as renal hypoplasia, one of the most common CAKUT disorders, which predisposes adult-onset hypertension and chronic kidney disease [31]. In addition, the microphotographs captured with the TEM revealed that *yotari* mice exhibited prominent podocyte damage with foot process effacement and a lack of filtration slits. After the injury, podocytes undergo the process of effacement in which they lose their structure, leading to a reduction in their filtration barrier function [32]. All forms of nephrotic syndrome, as well as focal segmental glomerulosclerosis (FSGS), are characterized by defects in podocyte structure or function [33].

Our current study showed the highest expression of DAB1 at the apical and lateral parts of cell membranes of the DCT, while REELIN was mostly scattered throughout the cytoplasm of the PCT. At the investigated time points, no morphological changes were observed in tubuli of *yotari* mice, but further investigation is necessary to elucidate the role of DAB1 in tubulointerstitial compartments. There was an occasional colocalization of the DAB1 and REELIN proteins, mostly in the DCT. These findings are following our previous work concerning the expression of these two proteins during fetal human kidney development [9]. This may suggest that DAB1 and reelin have similar roles in human and mouse kidneys by activating some of the downstream pathways, such as Crk, MAPK and PI3K/Akt/mTOR signaling cascades [34,35,36]. Our data also revealed increased reelin expression in the glomeruli of *yotari* mice. Interestingly, a previous study has shown that DAB1 phosphorylation and increased reelin expression in the glomeruli are accompanied by hypertension, proteinuria, and podocyte injury in the Ang II-infused rats [10].

In addition, immunofluorescence staining revealed an increased expression of NOTCH2 receptors in the glomeruli of P14 *yotari* mice. It is well-known that NOTCH2 expression is downregulated once nephron maturation is achieved, except in the conditions of renal injuries, such as diabetic nephropathy and FSGS [15,37]. Mice with the Adriamycin-induced nephrotic syndrome also showed increased expression of activated NOTCH2, which ameliorates fibrosis [38]. At the investigated time points, we did not notice increased fibrosis, but it remains to elucidate the exact role of the expressed NOTCH2 in the postnatal *yotari* kidneys. Further observation into whether fibrotic changes occur in the later stages would be necessary. However, there are already some findings that suggest the short-term effect of increased NOTCH2 activation is associated with a strong survival benefit for injured podocytes, which is lost in long-term models, such as diabetic nephropathy [39].

Higher LC3B expression in the glomeruli of P11 and P14 *yotari* mice probably reflected an accumulation of the autophagosomes in the podocytes. To clearly show that autophagy was accelerated. It is necessary to analyze the ratio of LC3-II and LC3-I protein expressions. Under normal conditions, autophagy is necessary for normal kidney function [40], especially in podocytes. Because of its limited capacity for cell division and replacement, they exhibit a high basal level of autophagy [41]. Despite these findings, elevation in LC3B expression has been reported in several glomerular diseases [42,43,44], mainly in association with foot process effacement and nephrotic syndrome development, as seen in prorenin receptor conditional knockout mice [45,46]. All these studies suggest a protective role of enhanced autophagy in pathological conditions, further supported by the findings of Adriamycin-induced nephropathy, where autophagy is activated to protect against podocyte injury [43], as well as in age-dependent glomerular disease where it delays the progressive functional decline of the kidney function [40]. Analysis of human kidney biopsies also showed evidence of increased autophagy in association with foot process effacement in several glomerular diseases, including minimal change nephrotic syndrome (MCNS), which is one of the most common causes of idiopathic nephrotic syndrome [19]. In the conditions of diabetic nephropathy and lipopolysaccharide-induced acute kidney injury, enhancing autophagy with some therapeutics through inhibition of the PI3K/AKT/mTOR pathway ameliorates kidney function [47,48]. All these findings implicate that autophagy has an indispensable cytoprotective role for stress adaption in kidney injury, and its modulation might be a promising therapeutic strategy, although, under some circumstances, autophagy can also be deleterious and progress into cell death.

Except for the elevation in autophagy, an increased level of apoptosis could also be observed in the glomeruli of P14 *yotari* mice. Autophagy usually prevents apoptosis, but under specific pathological circumstances, it can also have an opposite effect on cell survival by enhancing and promoting apoptosis [42]. After birth, apoptosis in the kidneys is greatly diminished, except in the conditions of kidney injury, such as idiopathic nephrotic syndrome where contributes to developing the disease [23].

A homozygous *Dab1^−/−^* (*yotari)* mice exhibited renal hypoplasia, revealing *Dab1^−/−^* as a potential novel cause of autosomal recessive congenital anomalies of the kidney and urinary tract. However, the main role of DAB1 during kidney development remains unclear. In addition, these mice displayed foot process abnormalities and an increased level of reelin, NOTCH2, LC3B and cleaved CASP3 proteins in the glomeruli that may lead to glomerular injuries, like nephrotic syndrome, which in correlation with hypoplasia, may be a potential cause of death of these animals during the weaning period. To confirm this hypothesis, it is necessary to provide additional information concerning blood pressure and other clinic-laboratory parameters, such as levels of serum creatinine, albumin, and proteins.

## Figures and Tables

**Table 1:** Antibodies used for immunofluorescence.

Primary Antibodies		Secondary Antibodies	
Type and Supplier	Dilution Ratio	Type and Supplier	Dilution Ratio
Rabbit polyclonal anti-Dab1 (ab 78200, Abcam, Cambridge, UK)	1:400	Alexa Fluor 488 AffiniPure donkey polyclonal anti-rabbit IgG (Jackson IR, 711-545-152)	1:400
Mouse monoclonal anti-reelin (sc 25346, Santa Cruz Biotechnology, Dallas, USA)	1:70	Rhodamine RedTM-X AffiniPure donkey anti-mouse IgG (Jackson IR, 715-295-151)	1:400
Rabbit polyclonal anti-Notch2 (ab 8926, Abcam, Cambridge, UK)	1:200	Alexa Fluor 488 AffiniPure donkey polyclonal anti-rabbit IgG (Jackson IR, 711-545-152)	1:400
Rabbit polyclonal anti-LC3B (ab 48394, Abcam, Cambridge, UK)	1:300	Alexa Fluor 488 AffiniPure donkey polyclonal anti-rabbit IgG (Jackson IR, 711-545-152)	1:400

**Table 2:** Staining intensity of specific antibodies in the kidneys of yotari and wild-type mouse in 4P, 11P and 14P. +++—strong reactivity; ++—moderate reactivity; +—mild reactivity; −—no reactivity, n/a—not applicable; G—glomeruli, PCT—proximal convoluted tubules, DCT—distal convoluted tubules, *P—*days of postnatal development.

		Antibody											
Postnatal Day (*P*)	Animal	REELIN			DAB 1			NOTCH2			LC3B		
		G	PCT	DCT	G	PCT	DCT	G	PCT	DCT	G	PCT	DCT
4	yot	++	+++	+	n/a	n/a	n/a	+	+	+	+++	++	++
	wt	−	+++	+	−	+	+++	++	+	+	++	+	++
11	yot	−	++	+	n/a	n/a	n/a	+	+	++	++	+	++
	wt	+	++	−	+	+	+++	+	+	++	++	+	++
14	yot	++	++	+	n/a	n/a	n/a	++	++	+++	++	+	++
	wt	+	+	−	+	+	+++	+	+	++	++	+	++

## Data Availability

Not applicable.
